# Molecular characterization of multidrug resistant (MDR) clinical isolates of *Pseudomonas aeruginosa* from Nsukka, South Eastern Nigeria

**DOI:** 10.4314/ahs.v23i3.57

**Published:** 2023-09

**Authors:** Martina C Agbo, Kenneth O Ugwu, Boniface N Ukwah, Ifeoma M Ezeonu

**Affiliations:** 1 Department of Pharmaceutical Microbiology and Biotechnology, University of Nigeria, Nsukka; 2 Department of Microbiology, University of Nigeria, Nsukka; 3 Department of Medical Lab. Science, College of Health Sciences, Ebonyi State University, Abakaliki, Nigeria

**Keywords:** MDRPA, PCR-RFLP, RAPD, Sequencing, 16S rRNA gene

## Abstract

**Background:**

*P. aeruginosa* is an important nosocomial pathogen with increasing resistance to antibiotics. Objective: This study was performed to evaluate the genetic relatedness of MDR clinical isolates of *P. aeruginosa*.

**Method:**

A total of 1000 samples were analysed in the study. Antibiotic resistance profiles of the isolates were determined using Kirby Bauer disk diffusion method. Polymerase chain reaction (PCR) and sequencing were simultaneously used to detect the consensus region of 16S rRNA. Genetic relatedness of the isolates was determined using restriction patterns from ALU 1 digest and random amplified polymorphic DNA.

**Results:**

Out of the 192 *P. aeruginosa* isolates recovered, 136 (78.83%) were multidrug resistant. Sequence analysis of the confirmed isolates (80.68%) revealed that all the isolates shared homology with each other and also showed sequence similarity to known strains of *P. aeruginosa* (ATCC 27853; KT 315654; KU 321274 and KT894767). The PCR-Restriction fragment length polymorphism (PCR-RFLP) analysis revealed that there was a lot of genetic relatedness among the isolates. The RFLP finger printing technique detected seven distinct RFLP types among the isolates.

**Conclusions:**

Thus, study shows that there is high prevalence of MDRPA and high degree of genetic relatedness among the MDRPA isolates circulating in Nsukka area.

## Introduction

*P. aeruginosa* has emerged as one of the most problematic nosocomial pathogens. It is considered as a major opportunistic pathogen that causes infection in immune depressed individuals, burn patients or cystic fibrosis patients 1.

It is responsible for various nosocomial infections, including those of the blood, lungs, wound, ear and urinary tract 2, 3. These infections are often difficult to eradicate due to the resistance of *P. aeruginosa* to many antibiotics 4. It is ubiquitous in nature and its ability to survive in moist environment with innate resistance to many antibiotics and antiseptics enable it to constitute a common pathogen in hospitals, particularly in Intensive Care Units (ICUs). The increasing use of antibiotics and the growing numbers of invasive procedures, together with the development of intrinsic and acquired resistance mechanism of *P. aeruginosa*, cause the evolution of numerous multidrug resistant *P. aeruginosa* (MDRPA) outbreak in clinical settings 5. Multidrug resistant *P. aeruginosa* is a public health problem that affects many countries of the world. A rapid and accurate system for the identification of *P. aeruginosa* is important to isolate patients and prevent further spreading of the diseases. Conventional biological typing methods such as biotyping, phage typing, serotyping and bacteriocin (pyocin) are well established and have been applied to the identification of *P. aeruginosa* infections but may lack discriminatory power and stability. Many studies are satisfied by using the API 20 test system or classical biochemical test for bacterial identification 6.

However, *P. aeruginosa* adaptive ability causes difficulties for the sensitivity of these methods. Therefore, it has become necessary to develop genotype-based characterization systems capable of accurately identifying these bacteria despite any phenotypic modifications. Molecular identification eliminates the problem of variable phenotype and allows for more accurate identification of bacteria 7. 16S rRNA genes are highly conserved among all organisms and they possess various unique species-specific regions that allow for bacterial identification. Polymerase Chain Reaction (PCR) is highly sensitive, specific and rapid method which vastly improved the detection of *P. aeruginosa* especially when using species-specific primer for 16S rRNA 8. Sequencing of 16S rRNA is a molecular technique for characterization of bacteria and tools used to analyse the phylogenetic relationship of an organism because of their information content, conservative nature, and universal distribution 9.

The use of DNA-based typing methods is becoming increasingly important in epidemiological survey and differentiation of Pseudomonas species. Several molecular typing techniques such as multilocus sequence typing, DNA microarray and indirect method of sequence analysis such as Restriction Fragment Length Polymorphism (RFLP); Random Amplified Polymorphic DNA (RAPD) and Pulsed Field Gel Electrophoresis (PFGE) have been described to differentiate between the isolates and clonal groups of *P. aeruginosa* 10, 11. Restriction fragment length polymorphism refers to the polymorphic nature of the locations of restriction enzyme site within defined genetic regions. Specific genetic loci are routinely amplified and examined for differences indicative of strain variation. The specific locus to be examined is amplified with gene-specific primers and is subjected to RFLP analysis. It is a reliable and relatively simple method but prior knowledge of the DNA sequence is necessary. On the other hand, Random Amplified Polymorphic DNA (RAPD) does not require any specific knowledge of the target DNA sequence, making it a flexible and powerful tool with general applicability.

These typing techniques (RFLP & RAPD) are useful for establishing clonal relationship between individual isolates in hospital settings and are therefore important to recognize nosocomial transmission and guide infection control practice. These molecular typing techniques have been widely used in developed countries. However, there is scanty information on the genetic study of *P. aeruginosa* in Nigeria, particularly in Enugu state. This study therefore aimed to isolate MDR clinical isolates of *P. aeruginosa*; characterize using PCR and sequencing and to elucidate their genetic relatedness using PCR-RFLP and RAPD.

## Materials and Methods

### Specimen Collection and Bacterial Identification

A total of 1000 samples comprising 950 clinical samples and 50 samples from hospital fomites were collected from various hospitals in Nsukka, South Eastern Nigeria between March and October, 2019. Of the 950 clinical samples, 36.84% (350) were wound/pus; 31.58% (300) were urine; 21.05% (200) were Ear swabs and 10.53% (100) were sputum. The study was approved by the Ethics Committee of the Faculty of Pharmaceutical Sciences, University of Nigeria, Nsukka. (ECC reference no: FRSRE/UNN/19/0008) and an informed consent was obtained from patients. Wound/pus swabs, ear swabs, sputum and swabs from hospital fomites were inoculated on brain heart infusion broth (Oxoid, UK) incubated for 24 h at 37^0^C. The next day, a loopful of the broth culture of each sample was inoculated onto sterile *Pseudomonas* cetrimide agar (Oxoid, U.K) which was supplemented with 10 ml/L of glycerol and incubated at 370C for 24 h. Urine samples were inoculated directly onto *Pseudomonas* cetrimide agar plate. Colonies were examined for the presence of blue-green or yellow-green pigments. Thereafter, the isolates were characterized by standard microbiological methods using *P. aeruginosa* ATCC 27853 as a control strain.

### Genomic DNA Extraction

The genomic DNA extraction was performed using zymo research fungal/bacterial DNA mini prep kit (Zymo Research, USA) according to the manufacturer's protocol. The purity of the extracted DNA was determined by checking the absorbance at 260 and 280 nm using Nanodrop Spectrophotometer.

### DNA Amplification

The extracted DNA was amplified with 16S rRNA primer targeting *P. aeruginosa* consensus region (Inqaba Biotechnical Company, South Africa) (Table 1). The Polymerase Chain Reaction (PCR) reaction was carried out using the New England Biolabs one Taq 2X master mix with standard buffer. The PCR reaction mixture was prepared in a 25 µl reaction volume containing 12.5 µl of 1X Master mix with standard buffer, 20 mM Tris-Hcl, 1.8 mM MgCl_2_, 22 mM NH_4_Cl, 22 mM KCl, 0.2 mM DNTPS, 5% glycerol, 0.06% GEPAL CA-630, 0.05% Tween 20, 25 units / ml Taq DNA polymerase (BioLab, England), 0.5 µl (10 µM) each of the forward and reverse primers (Inqaba biotech, South Africa), 5 µl of the extracted DNA and 6.5 µl of sterile Nuclease-free water (Norgen biotech corp. Canada). This was vortexed at low speed and placed in a thermal cycler (BIBBY) – Scientific Ltd, UK.

**Table 1:** Primer Sequences and PCR Conditions Used

Primer pair target	Primer Sequence (51-3^1^)	PCR Conditions	Amplicon size (bp)	References
16S rRNA gene (Consensus region)	F: AGAGTTTGATCCTGGCTCAG R: ACGGCTACCTTGTTACGCTT	Initial denaturation of 94^O^C for 5 min; 35 cycles of denaturation of 94^O^C for 1 min; annealing at 50^O^C for 1 min; extension at 72^O^C for 1 min and final extension at 72^O^C for 5 min.	1499	[12]
Universal primer target 16S rRNA	27F:AGAGTTTGATCCTGGCTCAG 1492R:GGTTACCTTGTTACGACTT	Initial denaturation of 95^O^C for 5 min;35 cycles of denaturation of 95^O^C for 1 min; annealing at 61^O^C for 30 s; extension at 72^O^C for 90 s and final extension at 72^O^C for 10 min.	1500	[13]
M13	TTATGTAAACGACGGCCACT	Initial denaturation of 94^O^C for 5 min;40 cycles of denaturation of 94^O^C for 1 min; annealing at 35^O^C for 1 min; extension at 72^O^C for 2 min and final extension at 72^O^C for 5 min.		[14]
B-01 B-11	TGCGCCCTTC GTAGACCCGT	Initial denaturation of 94^O^C for 2 min; 35 cycles of denaturation of 94^O^C for 1 min; annealing at 50^O^C for 1 min; extension at 72^O^C for 1 min and final extension at 72^O^C for 5 min		[15]

The PCR amplification program for the primers used is shown in Table 1. The PCR products were resolved on 1.5% agarose gel, stained with ethidium bromide (0.5 µg/ml) and electrophoresis was carried out at 70 volts for 90 min and visualized under UV transilluminator. A 100 bp DNA ladder (Norgen Biotek Corp, Canada) was used as DNA molecular weight marker.

### DNA Sequencing

The PCR amplicons of 16S rRNA primer were subjected to DNA sequencing by Inqaba Biotech Sequencing Service, South Africa. The sequencing was done with the ABIV 3.1 Big dye kit according to the manufacturer's instructions (http://mvz.berkeley.edu/egl/inserts/Big-DyeV3.1Protocolmannualpdf.) and the labelled products were then cleaned with zymoseq clean-up kit http://www.zymoresaerch.com/downloads/di/file/id/52/do452i.pdf.protocol. The eluted DNA samples (cleaned DNA products) were loaded on the Applied Bio systems(AB1 3500 ^X^ L sequencer with a 50 cm array, using Pop 7.The resulting sequences were aligned and compared with those stored in Gene Bank (http://www.ncbi.hlm.nlh.gov/genbank) using BLAST alignment software. Phylogenetic analysis of the sequenced DNA was performed using Qiagen work bench 7.5.1, Un-weighted pair group method using arithmetic average, distance measure and Bootstrap using Kimura 80 and 1000 replicates bioinformatics software respectively.

### PCR Restriction Fragment Length Polymorphism (PCR-RFLP)

Restriction enzyme analysis was carried out on the amplified PCR products using the restriction enzyme ALU 1 (Fast Digest, Thermo scientific) according to manufacturer's instruction. The reaction was carried out in a 30 µl reaction mixture containing 17 µl sterile deionised water; 2 µl of 10X Fast Digest Green buffer; 1 µl of the restriction enzyme ALU 1 and 10 µl of the PCR product. The mixture was then incubated at 65^0^C in a dry block for 5 min. Agarose gel electrophoresis was used to separate the digested DNA after incubation. The samples were separated on 1.5% agarose gel, stained with ethidium bromide (0.5 µg/ml) and electrophoresis was carried out at 80 volts for 90 min and visualized under UV transilluminator. A100 bp DNA ladder (Solis Biodyne) was used as DNA molecular weight standard.

### Random Amplified Polymorphic DNA (RAPD-PCR) Assay

The extracted DNA were submitted to RAPD genotyping using three primers (M13, B-01 and B-11) as shown in table 1. The PCR reaction was carried out using the New England Biolabs one Taq 2X master mix with standard buffer. The PCR reaction mixture was prepared in a 25 µl reaction volume containing 12.5 µl of 1X Master mix with standard buffer, 20 mM Tris-Hcl, 1.8 mM MgCl2, 22 mM NH4Cl, 22 mM KCl, 0.2 mM DNTPS, 5%glycerol, 0.06% GEPAL CA-630, 0.05% Tween 20, 25 units / ml Taq DNA polymerase (BioLabs, England), 0.5 µl (10 µM) each of each primer (Inqaba biotech corp, South Africa), 5 µl of the extracted DNA and 7 µl of sterile Nuclease-free water (Norgen biotech corp Canada).The PCR was performed in a thermal cycler machine (BIBBY)- Scientific Ltd, UK. The PCR amplification programs for the three primers used is shown in Table 1. The PCR-amplified products were separated on 1.5% agarose gel stained with ethidium bromide and electrophoresis was carried out at 70 v for 2 h. A 100 bp DNA ladder (Norgen Biotek Corp; Canada) was used as DNA molecular weight marker.

### Analysis of PCR- Restriction Fragment Length Polymorphism (PCR-RFLP) and Random Amplified Polymorphic DNA (RAPD-PCR)

Numerical Taxonomy System for Personal Computer (NTSYS-pc) v.2.20 software was used to analyse the band profiles. Dendrogram for cluster analysis of the strains were constructed using the Un-weighted pair group method using arithmetic average (UPGMA)

## Results

**Figure 1:**
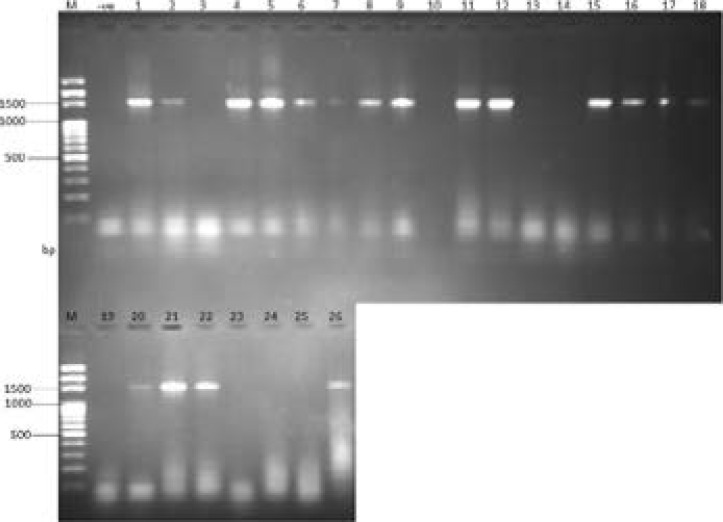
Agarose gel electrophoresis of PCR products of 16S rRNA primers used for restriction fragment length polymorphism (RFLP). Lane M= 100bp DNA ladder, lanes 1-2, 4-9, 11, 12, 15-18, 20-22 and 26 showed positive amplification for 1500 bp of the 16S rRNA gene lane 20 represent ATCC27853

Out of 1000 samples collected, *P. areugionsa* was recovered in 19.20% (192), comprising 136 (78.85%) multidrug resistant strains. The percentage occurrence of P. *areugniosa* isolates from different clinical samples was highest in wound/pus (32.86%); followed by ear swabs (22.50%); sputum (12.00%); hospital fomites (8.00%) and urine (5.00%). Analysis of the DNA purity using the ratios of absorbance at 260 and 280nm (A_260_/A_280_) showed relatively pure DNA samples with A_260/280_ values between 1.85 and 1.93. Quantitative analyses showed that the samples contained about 134-645 ng/µl DNA. The result of the amplification using the primer pair targeting the consensus region of bacterial 16S rRNA gene are presented in Figures 1.

**Figure 2:**
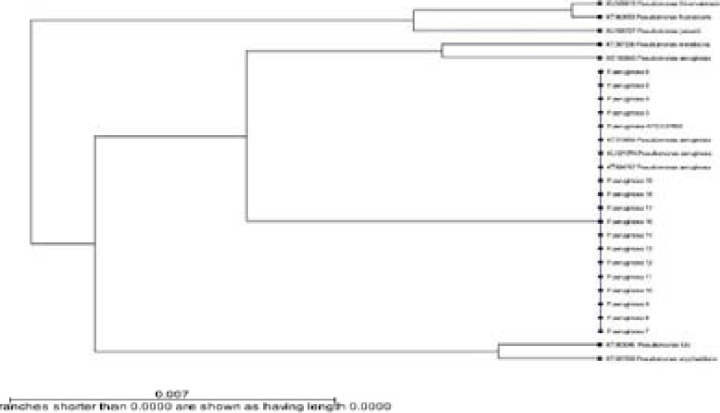
Phylogenetic tree showing 100% similarity among the *P. aeruginosa* isolates and comparison by the other *Pseudomonas* strains based on their 16SrRNA sequence

Approximately 80.68 % of the *P. aeruginosa* isolates expressed positive amplification of the 1499 bp of 16S rRNA gene, which confirmed the assumption that they were strains of *P. aeruginosa*. The 16S rRNA gene region of these isolates were sequenced, aligned, and blasted and sequences analysis revealed that all the isolates shared homology with each other and also showed sequence similarity to known strains of *P. aeruginosa* (Figure 2).

**Figure 3:**
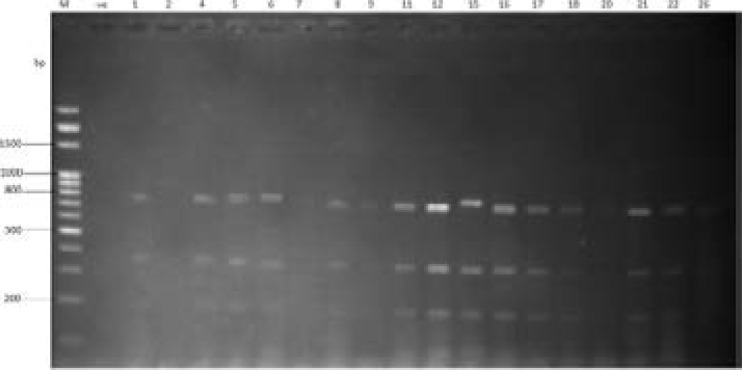
Agarose gel electrophoresis of PCR-RFLP (restriction fragment length polymorphism) using ALU 1 restriction digest. Lane M= 100bp DNA ladder

**Figure 4:**
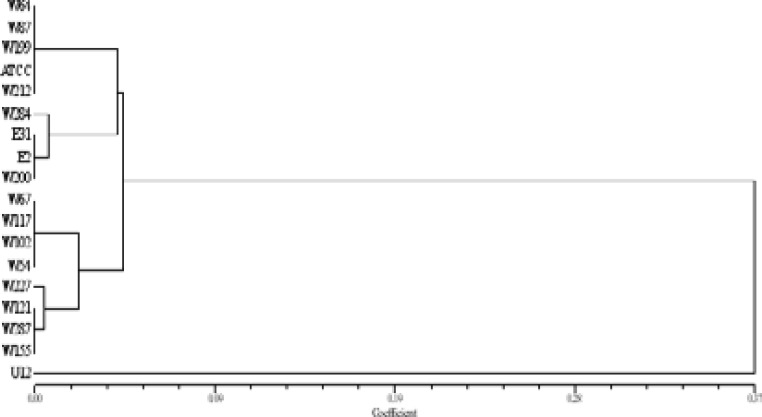
Dendrogram generated from the PCR-RFLP showing genetic relatedness among MDRPA isolates. W=wound; E=Ear swabs; and U= Urine

**Figure 5:**
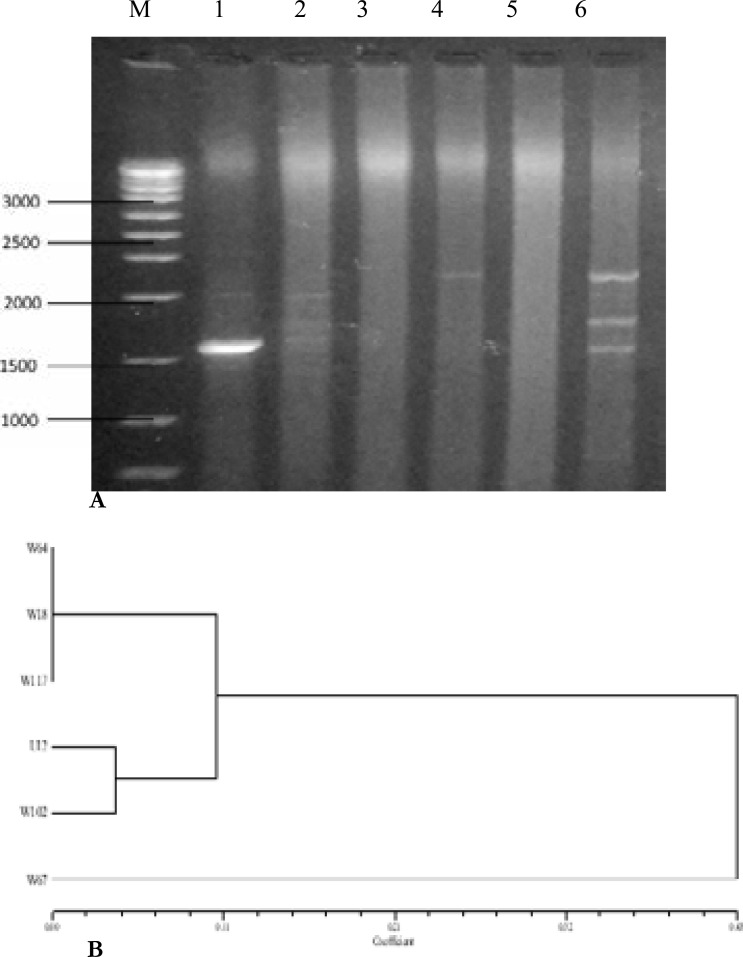
**(A)** Agarose gel electrophoresis of RAPD-PCR products after amplification using primer M13. Lane M= 1kb DNA ladder. **(B)** Dendrogram generated from the RAPD showing genetic relatedness among MDRPA isolates. W=wound; E=Ear swabs; and U= Urine

Agarose gel electrophoresis of DNA digested with ALU I revealed fourteen (14) restriction fragments with molecular weight ranging from 100 to 800 bp (figure 3). The RFLP finger printing technique detected seven (7) distinct RFLP types among 18 isolates (38.89% of polymorphisms) as presented in figure 4. Phylogenetic tree generated showed that there is a lot of genetic relatedness among the isolates. Out of three oligonucleotide primers (M13, B-01 and B-11) used for Random Amplified Polymorphic DNA (RAPD-PCR), only M13 was able to detect polymorphism. The result of the PCR revealed that out of eighteen (18) MDR *P. aeruginosa* isolates used, sample W64, U12, W18, W117, W102 showed DNA bands with molecular weight ranging from 1500 to 3000 bp as presented in figure 5. The dendrogram constructed grouped the isolates into two major clusters with three different RAPD types or genotypes (figure 5).

## Discussion

Identification of Pseudomonas creates a lot of difficulties 16, 17. Morphologically, similar species are alike to have biochemical characters. Sequence of highly conserved gene region 16S rRNA data helped us for the prediction of correct taxonomy. In this study, Polymerase chain reaction (PCR) was used to detect the *P. aeruginosa* consensus region of 16S rRNA gene with amplicon size of 1499 bp. This study revealed that 80.68% of the *P. aeruginosa* were confirmed by sequencing and PCR detection of 1499 bp amplicon of the 16S rRNA gene. This finding is consistent with the reports of some researchers who detected that some strains of MDRPA showed positive amplifications for the *P. aeruginosa* 16S rRNA gene 13, 18, 19. 16S rRNA gene sequence offered a useful method for the identification of bacteria. It had long been used as a taxonomic method in determining the phylogenies of bacterial species 7. Based on our finding the sequence analysis revealed that all the isolates shared homology with each other and showed sequence similarity to known strains of *P. aeruginosa (P. aeruginosa* ATCC 27853; KT315654 *P. aeruginosa*; KU321274 *P. aeruginosa* and KT894767 *P. aeruginosa*) as shown in figure 2. The present study agrees with the study carried out by Amutha and Kokila, who detected that after blast analysis all the *P. aeruginosa* isolates gave 99% similarity and the phylogenetic tree generated by NCBI tool proves that this organism is genetically related 20. Since determining bacterial genetic relatedness is essential for cross-infection evaluation, different genotyping methods have been established 21, 22.

In this study, PCR-Restriction fragment length polymorphism (PCR-RLFP) and Random amplified polymorphic DNA (RAPD) techniques were used to determine the genetic relatedness among multidrug resistant *P. areuginosa* isolates. PCR-Restriction fragment length polymorphism technique could be considered as a simple and sensitive method for detection and identification of bacteria. It has a high degree of discriminatory capability. In this study, the endonuclease digestion of 16S rRNA gene products generated different fragments ranging from 100 – 800 bp (Figure 3).

The analysis of the PCR-RFLP revealed that there was a lot of genetic relatedness among the isolates. The RFLP typing technique detected seven (7) clones/distinct RFLP types among the multidrug resistant isolates. Random amplified polymorphic DNA (RAPD -PCR) technique has been shown to be useful technique to study the genetic variability between bacterial strains including *P. aeruginosa* due to its great specificity and sensitivity 23. Moreover, RAPD-PCR could be considered as a source for tracking the infections since it is cost effective PCR based technique. In this study, DNA fingerprinting was performed using three oligonucleotide (RAPD) primers (M13, B-01 and B-11). It was observed that only the M13 primer was able to detect polymorphism among the isolates. The phylogenetic tree generated shows that these organisms are genetically related (figure 5).

The RAPD technique detected three (3) RAPD patterns. This finding is in line with the work of Husch who used the same M13 primer for RAPD-PCR fingerprinting for MDR *P. areuginosa* isolates obtained from intensive care Burn unit, and found that MDRPA isolates possessed identical RAPD patterns 14. Our results demonstrate that most of the isolates probably originated from the patients themselves; however, cross-infection of *P. areuginosa* between patients is possible to occur, suggesting nosocomial infection control problem.

## Conclusion

The present study, highlights that there is an alarming increase of clinical infections caused by multidrug resistant strain of *P. aeruginosa*. The PCR-RFLP and RADP analysis revealed that there was a lot of genetic relatedness among the isolates. The extensive use of broad spectrum of antimicrobial agents in various hospitals were probably responsible for the emergence and selection of this multidrug- resistant strains. This calls for rational drug use and need for improved personal and environmental hygiene especially within the hospital settings.
